# Controlling surface morphology of Ag-doped ZnO as a buffer layer by dispersion engineering in planar perovskite solar cells

**DOI:** 10.1038/s41598-024-55379-w

**Published:** 2024-02-26

**Authors:** Ghazaleh Bagha, Katayoon Samavati, Homam Naffakh-Moosavy, Laleh Farhang Matin

**Affiliations:** 1 Department of Physics, North Tehran Branch, Islamic Azad University, Tehran, Iran; 2 Department of Materials Engineering, Tarbiat Modares University (TMU), P.O. Box 14115-143, Tehran, Iran

**Keywords:** Buffer layer, Water–ethanol mixtures, Dispersion, ZnO, Electron transport layer, Optics and photonics, Physics, Materials science, Materials for devices, Materials for energy and catalysis, Materials for optics

## Abstract

In recent years, the power conversion efficiency (PCE (%)) of perovskite solar cells (PSCs) has improved to over 26%. To enhance the photovoltaic properties of PSCs, several materials for the electron transport layer (ETL) have been investigated. Zinc oxide (ZnO) is a significant ETL due to its high electron mobility and optical transparency in PSCs. As a result of various deposition methods, ZnO ETL can be processed at low temperatures. On the other hand, based on several studies, metal-doped ZnO can facilitate electron transfer, thereby improving the performance of un-doped ZnO ETL-based PSCs. Here, to improve the PCE (%) and long-term stability of un-doped ZnO ETL-PSCs, silver (Ag)-doped ZnO 1wt% as a buffer layer is examined. In this paper, with the addition of an organic solvent (ethanol) to the dispersion of Ag-doped ZnO 1 wt% nanoparticles (NPs) in deionized (DI) water, the morphology of the buffer layer (Ag-doped ZnO 1 wt%) can be controlled. This approach focuses on reducing the wettability of the ZnO/Ag-doped ZnO 1 wt% bilayer ETLs and enhancing the stability of un-doped ZnO ETL-PSCs. According to the results, the ZnO/H_2_O-ethanol mixtures-Ag-doped ZnO 1 wt% bilayer ETL leads to the formation of high-quality perovskite with low defects, reducing the recombination rate, and long-term stability of un-doped ZnO ETL-PSCs in ambient conditions.

## Introduction

Converting solar energy into electricity is one of the major challenges in the modern world. In recent years, the power conversion efficiency (PCE (%)) of perovskite solar cells (PSCs) has been reported between 3% to over 26%^1–3^. The significant improvement in PCEs (%) (over 26%) could be attributed to effective materials design, prevention of charge recombination, and optimizing interfaces in PSCs^4,5^. Nevertheless, the stability issue is a challenge that prevents large-scale commercial applications of PSCs. Recently, there have been many attempts to improve the stability of PSCs^2,6–8^. For instance, the inorganic cesium (Cs) cation has gained attention in triple-cation perovskites. The triple-cation perovskites containing Cs exhibit high thermal and moisture stability^9–13^. One of the most effective triple-cation mixed-halide perovskites to enhance light absorption and moisture resistance is Cs_0.05_(MA_0.17_FA_0.83_)_0.95_Pb(I_0.83_Br_0.17_)_3_^14–17^.

Furthermore, in another paper, the introduction of 1-methanethiol (PT) into the perovskite (FACsPbI_3_) inhibits bulk defects and stabilizes lead (Pb) ions. According to the results, thiol groups in PT are capable of stabilizing uncoordinated Pb ions and passivating iodine vacancies, which reduces nonradiative recombination and improves air stability in PSCs. Accordingly, the PT-modified inverted device exhibits a PCE (%) of 22.46%, which is higher than the control device (20.21%)^18^.

In addition, electron transport layers (ETLs) are crucial for high-performance PSCs. In general, planar or mesoporous structures of titanium dioxide (TiO_2_) are used as ETLs in PSCs, which block holes and extract charge carriers (electrons). Nevertheless, the preparation process of TiO_2_ ETL involves high temperatures and complex treatment processes that increase the production cost of TiO_2_ ETL-based PSCs^19,20^. Hence, other n-type materials have been studied to improve the photovoltaic properties of PSCs, including (SnO_2_, ZnO, WO_3_, and In_2_O_3_). Currently, zinc oxide (ZnO) is one of several ETLs that can be processed at low temperatures. The un-doped ZnO ETL has gained attention due to excellent light absorption, and high electron mobility in PSCs^21–24^. However, the ZnO ETL/perovskite interface exhibits poor chemical stability, which leads to limited progress compared with TiO_2_ and SnO_2_ ETLs-based PSCs. Numerous studies have been reported to reduce the instability problem for ZnO-based PSCs, such as metal doping and surface modification/passivation^25–29^. Moreover, the ZnO bilayer (ZnO with buffer layer) ETLs have garnered great interest due to the improvement of the ZnO ETL/perovskite interface. The ZnO bilayer ETL has various advantages, including defect passivation, faster electron extraction, improving interface contact between ETL and perovskite layer, energy-level tuning of ZnO ETL and perovskite, as well as minimizing/eliminating degradation of the perovskite layer^23,30^. For example, the ZnO/TiO_x_ ETL bilayer contributed to modifying the ZnO/perovskites interface and reducing the recombination rate. Finally, PCE (%) of ZnO/TiO_x_ ETL bilayer ETL-based PSCs showed over 19%^30^. Furthermore, metal-doped ZnO as a buffer layer reduces the recombination rate in ZnO-based PSCs. The results of one research indicate that AZO/ZnO bilayer ETL can extract charge carriers more than un-doped ZnO ETL^31^. On the other hand, for the deposition of ZnO ETL, numerous approaches have been studied, including dispersion-processed spin coating and spray pyrolysis. Among these approaches, spin-coating with the dispersion of nanoparticles (NPs) is preferred due to its ease of preparation, low-temperature processing, and low cost^32^. Moreover, due to the high temperature required by the annealing process (180 °C), it is difficult to achieve ZnO ETL with uniform grain size and a fully covered surface in PSCs^33^. Furthermore, the dispersion of ZnO NPs in deionized (DI) water ensures the regulation of grain size^34,35^. Hence, the ZnO layer usually exhibits some aggregation of NPs after the dispersion of ZnO NPs in DI water and filtering, which hinders performance enhancement in PSCs^35^.

In this paper, silver (Ag)-doped ZnO 1 wt% as a buffer layer is examined to increase PCE (%) and long-term stability in the un-doped ZnO-based PSC. It should be noted that different concentrations of silver doping (in the range of 1 wt% to 5 wt%) have been investigated the in the report of papers^35,36^. Silver doping concentration was optimized at 1 wt% for Ag-doped ZnO 1 wt% buffer layer. Furthermore, we have used the dispersion engineering process of nanoparticles (NPs) to precisely control the morphology of the Ag-doped ZnO 1 wt% layer and increase the performance of the un-doped ZnO-based PSC. In this approach, an organic solvent (ethanol) is added to the dispersion of nanoparticles (NPs) in deionized (DI) water. Based on the results, the Ag-doped ZnO 1 wt% layer was prepared by NPs dispersing in water (H_2_O)-ethanol mixtures, which led to the formation of a perovskite layer with low defects, reducing recombination rate, and long-term stability of un-doped ZnO ETL-PSCs in ambient conditions.

## Methods

### Synthesis and materials

This paper used a combustion synthesis (CS) method for the preparation of un-doped ZnO nanoparticles (NPs), emphasizing cost-effectiveness^37^. To ensure the purity of ZnO NPs, high-purity materials from reputable companies like Merck were utilized.

In this synthesis process, deionized water (DI) with a pH of 7.6 (the resistivity of DI water utilized was approximately 1.8 MΩ × cm) is used. High-purity zinc nitrate by glycine and glucose fuels were added to 20 ml (ml) of DI water. Then, silver nitrate (1 wt%) was added to the specimens.

On the other hand, the Cs_0.05_(MA_0.17_FA_0.83_)_0.95_Pb(I_0.83_Br_0.17_)_3_ perovskite was prepared using monovalent organic cations sourced from Dyesol, TCI, and abcr GmbH. This preparation involved dissolving FAI (1 M), CsI (0.05 M), PbI_2_ (1.1 M), MABr (0.2 M), and PbBr_2_ (0.22 M) in anhydrous dimethylformamide/dimethyl sulfoxide (4:1 v/v) solution^38^.

### Fabrication of ZnO ETL and buffer layer

Fluorine-doped tin oxide substrates (FTO with 10 Ω sq^–1^, Nippon Sheet Glass) with a surface area of 1.4 × 1.4 cm^2^ were cleaned using water, followed by acetone and ethanol for 15 min each, then treated with UV-ozone for 30 min (min).

The un-doped ZnO NPs were dispersed in DI water (1 mg in 1 ml) by sonication for 4 h (h), followed by 12 h of stirring at 1500 rpm. On the cleaned FTO substrates, un-doped ZnO ETLs were deposited by the spin-coating method at 3000 rpm for 30 s (s). To obtain uniform distributions of smaller un-doped ZnO particles, a filter was used to improve perovskite deposition. Hence, the dispersion of ZnO NPs was filtered by a 0.22 µm hydrophilic filter. Afterward, these specimens were annealed at 100 °C for 30 min. To prepare Ag-doped ZnO 1 wt% NPs as a buffer layer, DI water, and water (H_2_O)-ethanol mixtures (0.5:0.5 v/v) are used for dispersing of Ag-doped ZnO 1 wt% NPs. In both DI water (1 ml) and water (H_2_O)-ethanol mixtures (0.5:0.5 v/v), 1 mg of Ag-doped ZnO 1 wt% NPs were dispersed with sonication for 10 h and stirred at 1500 rpm for 12 (h). Next, Ag-doped ZnO 1 wt% NPs dispersion was filtered with a 0.22 µm hydrophilic filter. In this paper, various spinning speeds (at 3000 rpm, 3500 rpm, 4000 rpm, and 4500 rpm for 20 s) were used to deposit the Ag-doped ZnO buffer layer in the ZnO/Ag-doped ZnO 1 wt% bilayer ETL. The thickness of the Ag-doped ZnO 1 wt% buffer layer was optimized by the spin-coating method (at 4000 rpm for 30 s). After that, the specimens were annealed at 90 °C for 45 min.

### Fabrication of perovskite films, spiro-OMeTAD hole transporting layer (HTL), and conductive coating

**Figure 1:**
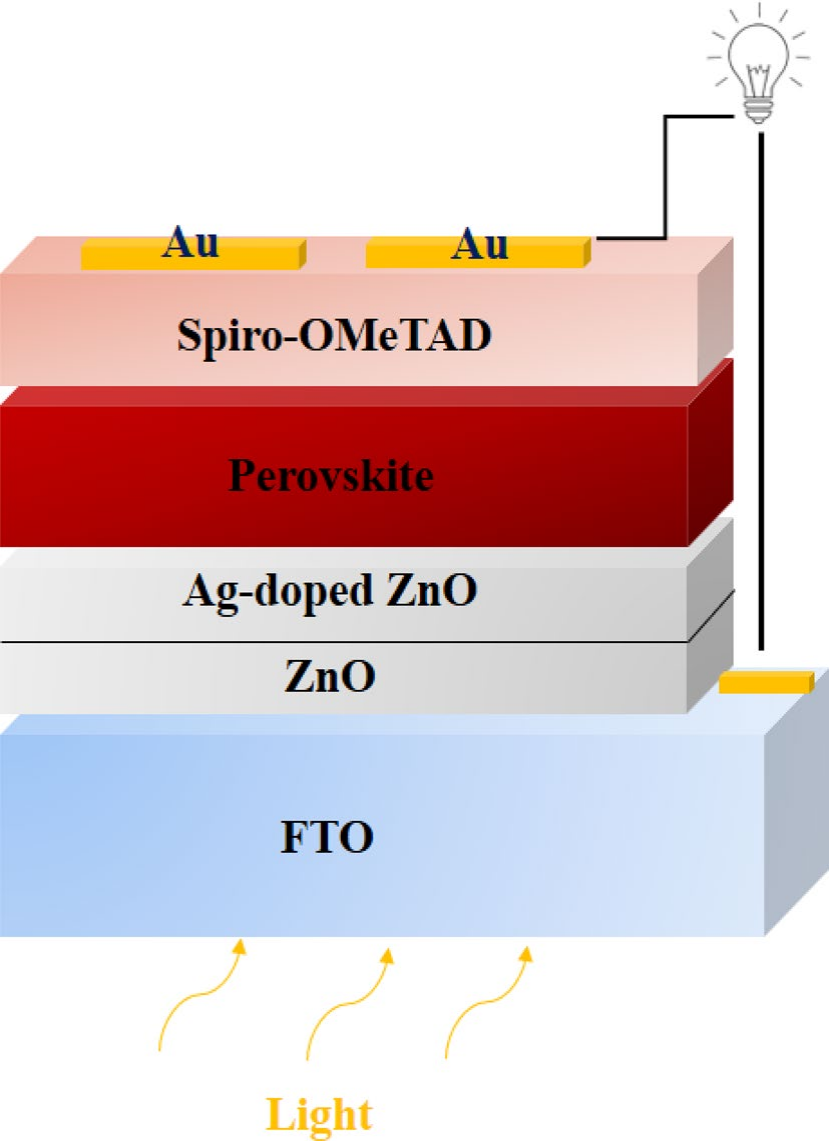
The structure of PSC in this paper.

In the report of papers^35,36^, described perovskite and spiro-OMeTAD deposition processes. On the spiro-OMeTAD HTL, a gold (Au) layer was deposited with a thickness of 60–80 nm by the PVD method^36,39^. Figure 1 shows the structure of PSC in this paper.

### Characterization and measurements

This paper examines the morphology of Ag-doped ZnO 1 wt% NPs dispersions in H_2_O-ethanol mixtures and H_2_O using X-ray diffraction (XRD). The XRD analysis was obtained X’Pert MPD- Philips (Anode material: Cu, (Reference code: 00-036-1451)). Moreover, to analyze Ag-doped ZnO 1 wt% NPs, transmission electron microscopy (TEM) was provided using (EM208S, Philips, and 100kV-Netherland). Elemental analyses of the Ag-doped ZnO 1 wt% NPs were undertaken using X-ray fluorescence (XRF). The XRF analysis was provided using (PHILIPS, PW1410). In this study, dry powdered samples of ZnO NPs and Ag-doped ZnO 1 wt% NPs were XRF analyzed in equal quantities (5 gr). Moreover, atomic force microscopy (AFM) and cross-sectional FE-SEM analyses were used to examine the surface morphology of both types of morphology for Ag-doped ZnO 1 wt% buffer layer in ZnO/Ag-doped ZnO 1 wt% bilayer ETL. For AFM images, a (Veeco-CPII) was used. Photoluminescence (PL) and ultraviolet–visible (UV–Vis) spectroscopy were used to examine the optical properties of Ag-doped ZnO 1 wt% as a buffer layer. The UV–visible analysis was performed using a (Thermo-AVATAR). Furthermore, PL analysis was carried out by (Avaspec 2048 TEC, Avantes, Netherlands). The surface morphology of the ZnO/Ag-doped ZnO 1 wt% bilayer was examined using field emission scanning electron microscopy (FE-SEM). The FE-SEM images were prepared using TESCAN’s MIRA III. ZnO/Ag-doped ZnO 1 wt% bilayer ETL-based planar PSC was evaluated using current–voltage (J-V) characteristics. The J-V characteristics of PSCs are determined under 100 mW·cm^−2^ AM 1.5G solar intensity. The J-V characteristics were prepared by (IRASOL, IV-28). Finally, external quantum efficiency (EQE), is measured for the PSCs. The EQE analysis is performed by (IRASOL, IPCE-020).

### Ethical approval

This work does not apply to both human and/or animal studies.

## Results

### XRF analysis of Ag-doped ZnO NPs

Table 1 shows the XRF analysis of un-doped ZnO NPs and Ag-doped ZnO 1 wt% NPs. In the XRF analysis, several oxides were detected, and their concentrations are represented as weight percentages in Table 1. Moreover, silver (Ag) atoms are reported as Ag_2_O in Table 1. In the XRF analysis, Ag and ZnO were confirmed as elements of Ag-doped ZnO 1 wt% NPs.

**Table 1:** XRF analysis of un-doped ZnO and Ag-doped ZnO 1wt% NPs.

Component	Un-doped ZnO (wt%)	Ag-doped ZnO (wt%)
Al_2_O_3_	–	–
SiO_2_	–	–
P_2_O_5_	–	–
K_2_O	–	–
CaO	–	–
CuO	–	–
ZnO	98.40	97.35
Ag_2_O	–	1.03

### Morphology analysis of Ag-doped ZnO NPs (FE-SEM and TEM)

**Figure 2:**
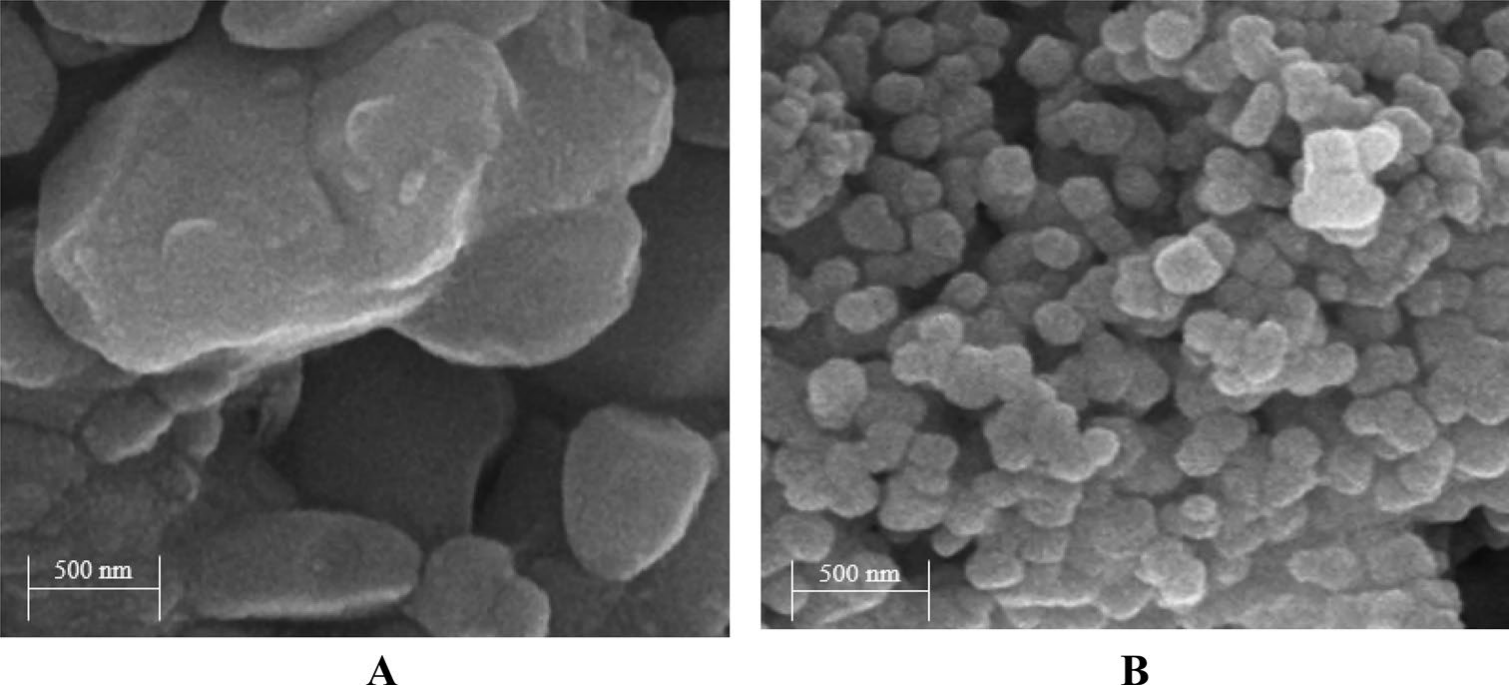
FE-SEM images of (**A**) un-doped ZnO NPs and (**B**) Ag-doped ZnO 1 wt% NPs.

FE-SEM images of un-doped ZnO NPs and Ag-doped ZnO 1 wt% NPs are displayed in Fig. 2A,B. The type of metals, the concentrations of metal doping, and the synthesis circumstances affect the size of NPs. As shown in Fig. 2A, the size of un-doped ZnO NPs is around 55–78 nm (nm). According to Fig. 2B, the Ag-doped ZnO 1 wt% NPs have a size of 33–44 nm and are virtually spherical. Based on the results, the size of un-doped ZnO NPs decreases after Ag doping for various reasons. One theory is that metal doping prevents crystallite growth^40^. Furthermore, metal doping may impact the surface energy and reactivity of NPs.

**Figure 3:**
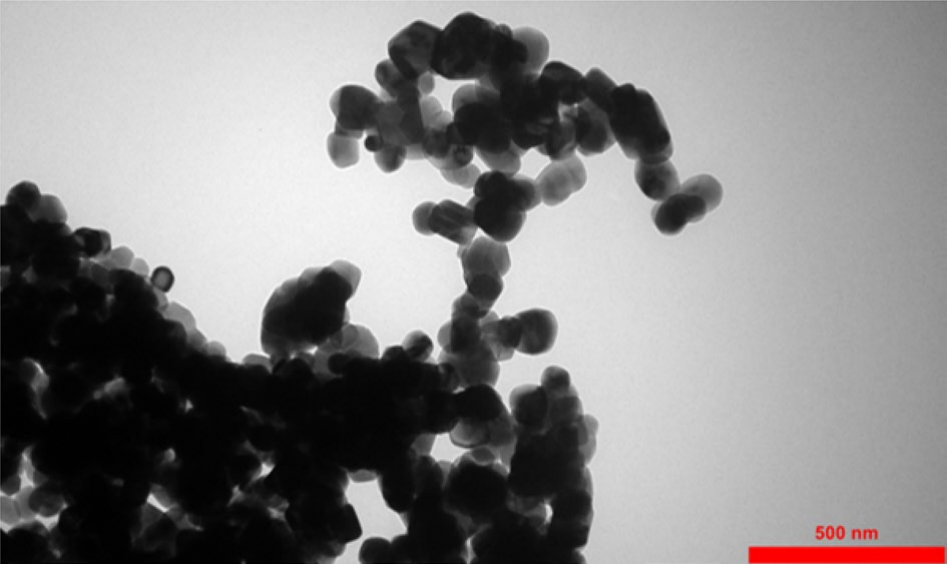
TEM images of Ag-doped ZnO 1 wt% NPs.

On the other hand, aggregation of Ag-doped ZnO 1 wt% NPs is illustrated in Fig. 2B. Aggregation of NPs can lead to defects, such as oxygen vacancies. The oxygen vacancies can significantly impact the optical properties and electrical conductivity. Moreover, Fig. 3 displays a TEM image of Ag-doped ZnO 1 wt%. In the TEM image, aggregation of Ag-doped ZnO 1 wt% NPs show more clearly than in the FE-SEM image.

It should be noted that various factors such as sample preparation can lead to differences in the size of Ag-doped ZnO 1 wt% NPs between TEM and FE-SEM analyses. This difference could be explained by the agglomeration of Ag-doped ZnO 1 wt% NPs in TEM analysis, which increases the size of NPs. In this paper, the TEM image of Ag-doped ZnO 1 wt% NPs is prepared after dispersing Ag-doped ZnO NPs in DI water, which can result in more agglomeration of NPs in Fig. 3.

### XRD analysis of bilayer ETLs

**Figure 4:**
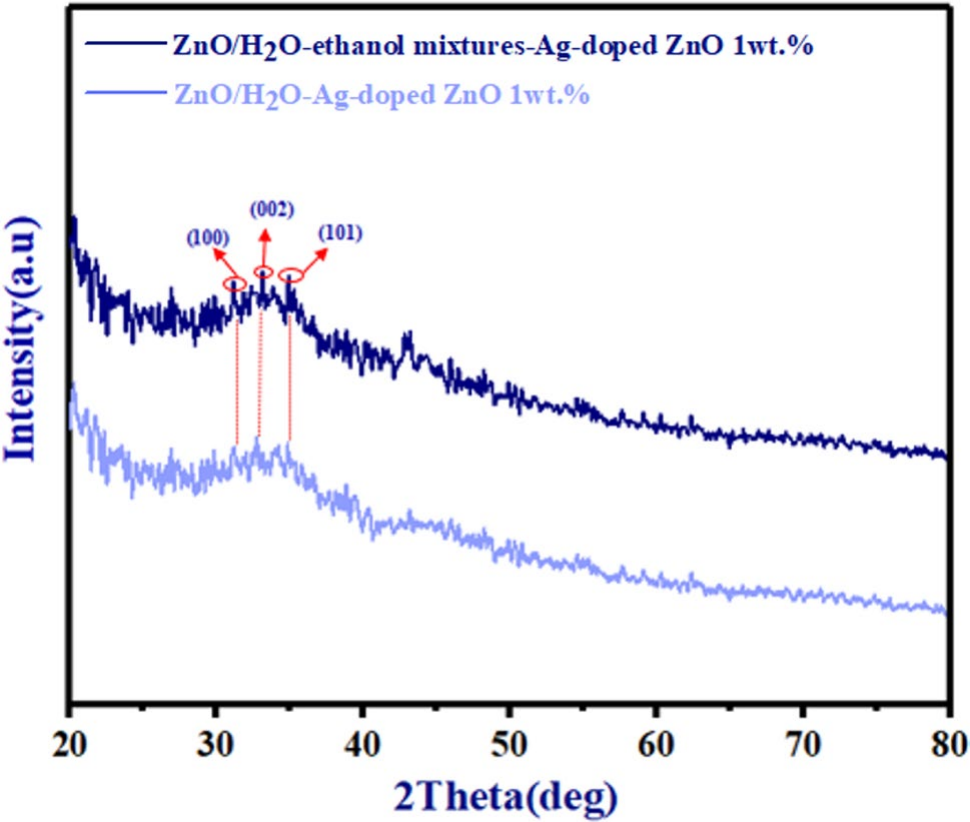
XRD patterns of both types of morphology for Ag-doped ZnO 1 wt% buffer layer in ZnO/Ag-doped ZnO 1 wt% bilayer ETL.

XRD spectra were evaluated to examine the variations in crystallinity of ZnO/Ag-doped ZnO 1 wt% bilayer ETLs on glass substrates with different dispersions of Ag-doped ZnO NPs in Fig. 4. Both types of morphology for Ag-doped ZnO 1 wt% buffer layer in ZnO/Ag-doped ZnO 1 wt% bilayer ETL exhibit typical diffraction peaks assigned to the (100), (200), and (101) planes. In comparison with ZnO/H_2_O-ethanol mixtures-Ag-doped ZnO 1 wt% bilayer, the XRD pattern of the ZnO/H_2_O-Ag-doped ZnO 1 wt% bilayer formed with shows lower peaks in Fig. 4. According to Fig. 4, the weaker peaks indicate uncontrolled random aggregation of the Ag-doped ZnO 1 wt% NPs and low-quality buffer layer quality. The intensity of peaks is depicted with red circles in Fig. 4.

### Surface morphology analysis of bilayer ETLs

#### AFM analysis

**Figure 5:**
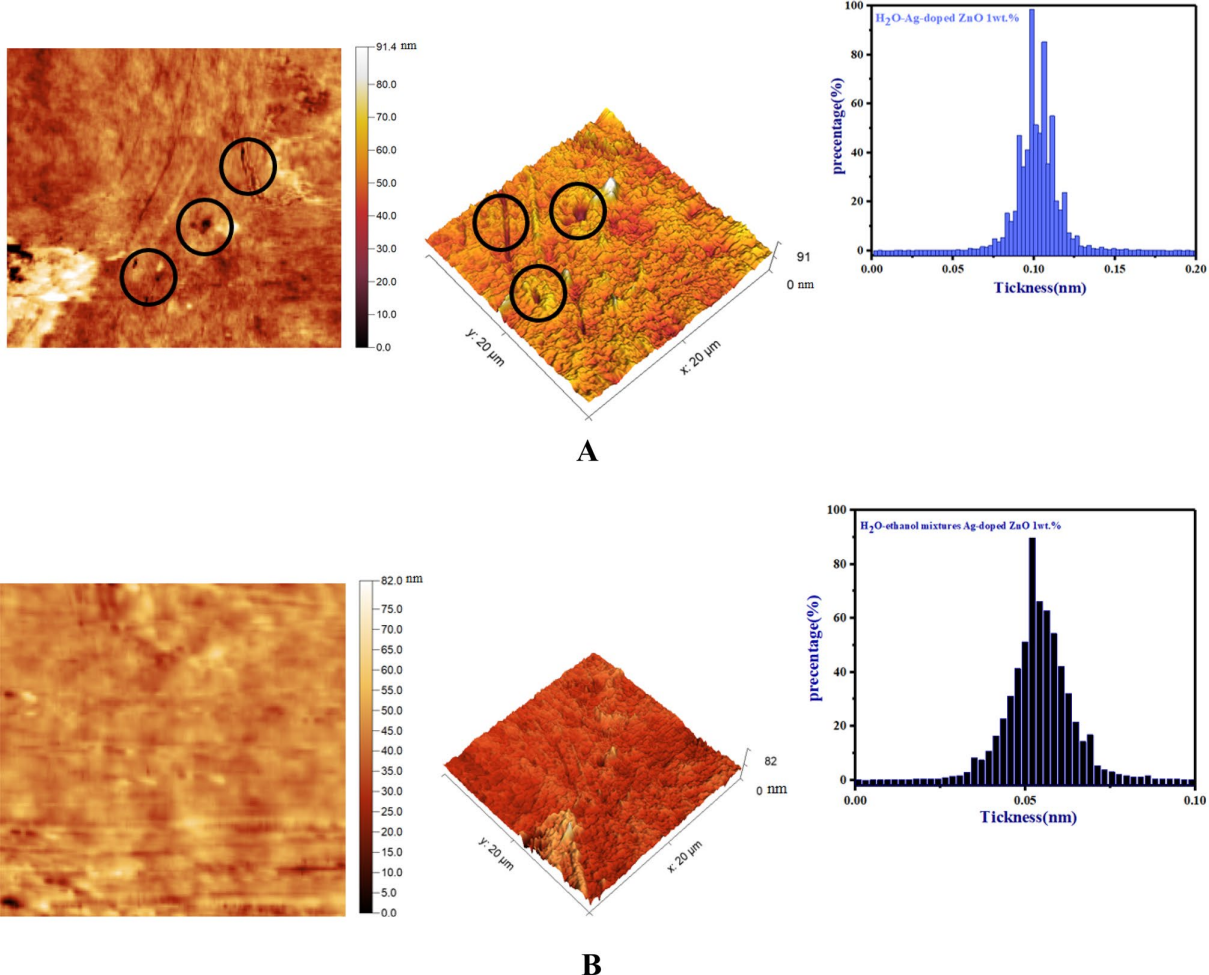
AFM images of (**A**) glass/ZnO/H_2_O-Ag-doped ZnO 1 wt%, and (**B**) glass/ZnO/H_2_O-ethanol mixtures-Ag-doped ZnO 1 wt% bilayer ETLs.

Figure 5A,B show 3D and 2D AFM images, and thickness histograms of ZnO/H_2_O-Ag-doped ZnO 1 wt% and ZnO/H_2_O-ethanol mixtures-Ag-doped ZnO 1 wt% bilayer ETLs respectively. Initially, ZnO/H_2_O-Ag-doped ZnO 1 wt% and ZnO/H_2_O-ethanol mixtures-Ag-doped ZnO 1 wt% bilayer ETLs were deposited by spin-coating method on glass substrates (after filtration).

As a result of the uniform distribution of smaller un-doped ZnO and Ag-doped ZnO NPs 1 wt% after filtration, the morphology of the perovskite layer improved.

Furthermore, Table 2 depicts root-mean-square roughness (R_ms_(nm)) and average roughness (R_a_(nm)) of ZnO/H_2_O-Ag-doped ZnO 1 wt% and ZnO/H_2_O-ethanol mixtures-Ag-doped ZnO 1 wt% bilayer ETLs respectively.

**Table 2:** Quantitative roughness parameters of ZnO/H_2_O-Ag-doped ZnO 1wt% and ZnO/H_2_O-ethanol mixtures-Ag-doped ZnO 1wt% bilayer ETLs.

Bilayer ETL	R_a_ (nm)	R_ms_ (nm)
ZnO/H_2_O-Ag-doped ZnO 1wt%	1.95	2.74
ZnO/H_2_O-ethanol mixtures-Ag-doped ZnO 1wt%	1.49	2.00

According to Table 2, the root-mean-square roughness (R_ms_(nm)) of the ZnO/H_2_O-Ag-doped ZnO 1 wt% bilayer ETL is 2.74. Moreover, based on Fig. 5A, the surface of ZnO/H_2_O-Ag-doped ZnO 1 wt% bilayer ETL reveals that substantial gaps exist between the un-doped ZnO NPs. In Fig. 5A, these gaps are represented by black circles.

On the other hand, compared to ZnO/H_2_O-Ag-doped ZnO 1 wt% bilayer ETL, the ZnO/H_2_O-ethanol mixtures-Ag-doped ZnO 1 wt% bilayer ETL shows a smoother surface in Fig. 4B.

In addition, the ZnO/H_2_O-ethanol mixtures-Ag-doped ZnO 1 wt% bilayer ETL has a lower R_MS_ value (2.00 nm) than the ZnO/H_2_O-Ag-doped ZnO 1 wt% bilayer ETL. Therefore, Ag-doped ZnO 1 wt% NPs dispersed in H_2_O-ethanol mixtures are effective at filling gaps between un-doped ZnO NPs in ZnO/H_2_O-ethanol mixtures-Ag-doped ZnO 1 wt% bilayer ETL. To further clarify the effects of H_2_O-ethanol mixtures-Ag-doped ZnO 1 wt% modification, Fig. 5A,B present thickness histograms of ZnO/H_2_O-Ag-doped ZnO 1 wt%, and ZnO/H_2_O-ethanol mixtures-Ag-doped ZnO 1 wt% bilayer ETLs respectively. Based on Fig. 5B, compared to the thickness histogram of ZnO/H_2_O-Ag-doped ZnO 1 wt% bilayer ETL in Fig. 5A, ZnO/H_2_O-ethanol mixtures-Ag-doped ZnO 1 wt% bilayer ETL has a lower thickness due to homogeneous surface morphology and reducing random aggregation of Ag-doped ZnO 1 wt% NPs. As a conclusion of the AFM findings, the H_2_O-ethanol mixtures-Ag-doped ZnO 1 wt% modification layer is effective in reducing surface defects of the un-doped ZnO ETL.

#### Cross-sectional FE-SEM of bilayer ETL/perovskite

**Figure 6:**
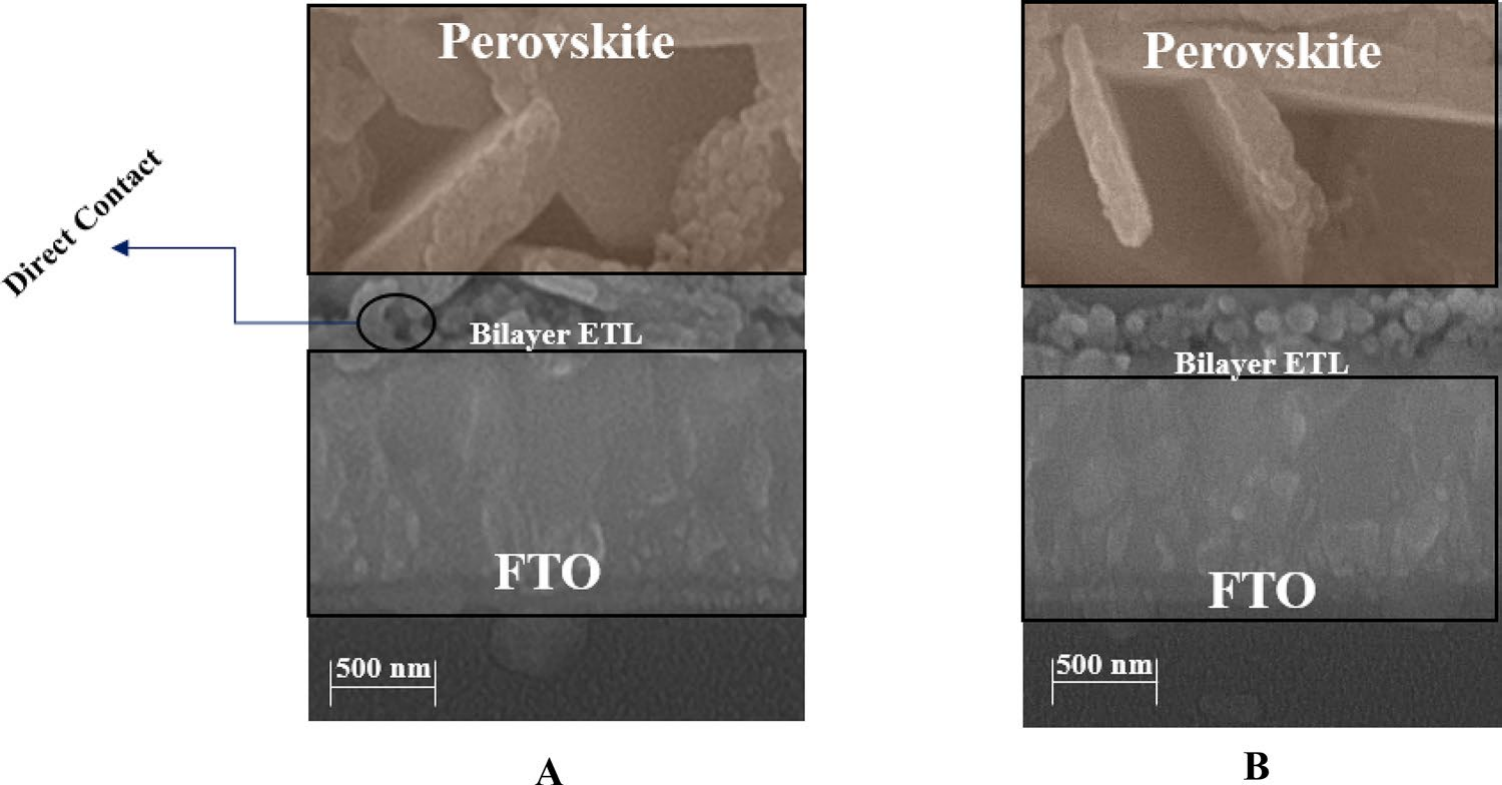
The cross-FESEM of the morphology of (**A**) FTO/ZnO/H_2_O-Ag-doped ZnO 1wt% bilayer ETL/perovskite, and (**B**) FTO/ZnO/H_2_O-ethanol mixtures-Ag-doped ZnO 1wt% bilayer ETL/perovskite.

Figure 6A,B indicate the cross-FESEM of the morphology of FTO/ZnO/H_2_O-Ag-doped ZnO 1 wt% bilayer ETL/perovskite and FTO/ZnO/H_2_O-ethanol mixtures-Ag-doped ZnO 1 wt% bilayer ETL/perovskite respectively. According to Fig. 6A, perovskite crystals stack on top of each other in irregular grain size with random defects, when H_2_O is used as a dispersion of Ag-doped ZnO 1wt% NPs in the ZnO/H_2_O-Ag-doped ZnO 1wt% bilayer ETL. Furthermore, based on Fig. 6A, the surface of the ZnO/H_2_O-Ag-doped ZnO 1wt% bilayer ETL is not completely covered by dense pores, which leads to the direct contact between the FTO and perovskite layer. As a result of direct contact between the FTO and perovskite layer in the ZnO/H_2_O-Ag-doped ZnO 1wt% bilayer ETL, the transportation of carriers is confined, and increasing recombination rate. In contrast, the pores of ZnO/H_2_O-ethanol mixtures-Ag-doped ZnO 1wt% bilayer ETL reduced when Ag-doped ZnO 1wt% NPs were dispersed in H_2_O-ethanol mixtures. In comparison with the ZnO/H_2_O-Ag-doped ZnO 1wt% bilayer ETL, by arranging the grains densely in the H_2_O-ethanol mixtures-Ag-doped ZnO 1wt% buffer layer, the ZnO/H_2_O-ethanol mixtures-Ag-doped ZnO 1wt% bilayer ETL surface appears smoother, thereby resulting in the formation of the perovskite layer with higher quality. Moreover, the perovskite layer was formed with low defects due to the reduced wettability of ZnO/H_2_O-ethanol mixtures-Ag-doped ZnO 1wt% bilayer ETL.

It should be noted that we used a filter for dispersion to achieve uniform NPs in the bilayer ETL, resulting in a reduced thickness of ZnO/H_2_O-ethanol mixtures-Ag-doped ZnO 1wt% bilayer ETL (approximately 82–91 nm). Moreover, the thickness of Ag-doped ZnO 1wt% is approximately 30–33 nm.

**Figure 7:**
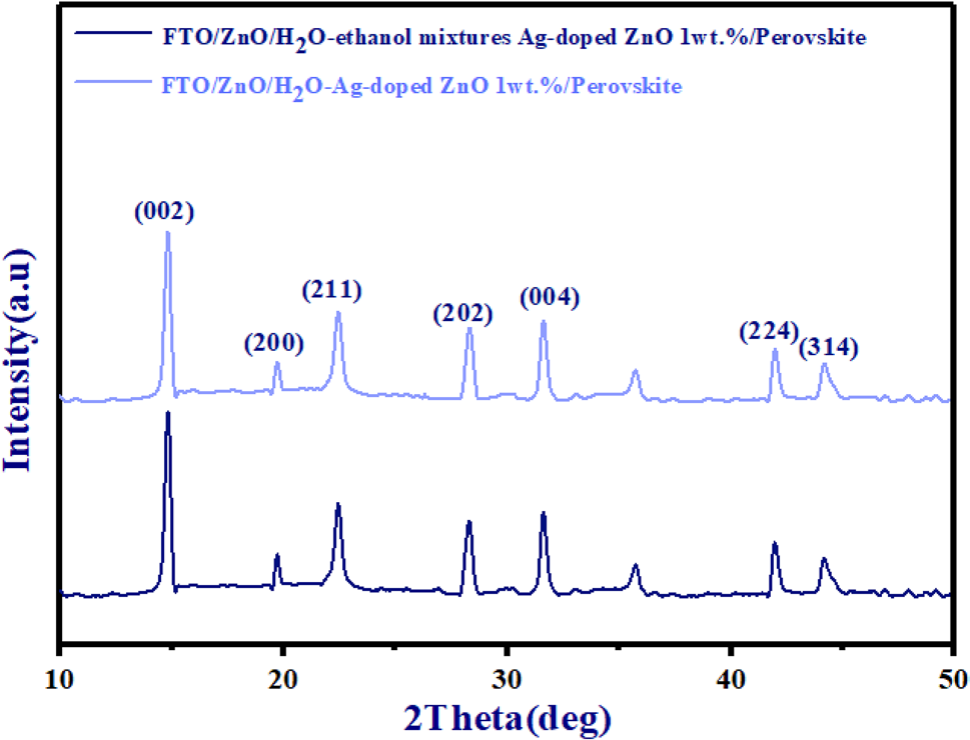
The XRD patterns of FTO/ZnO/H_2_O-Ag-doped ZnO 1wt%/perovskite and FTO/ZnO/H_2_O-ethanol mixtures-Ag-doped ZnO 1wt%/perovskite.

For further confirmation of the complete formation of the perovskite layer, Fig. 7 displays the XRD patterns of FTO/ZnO/H_2_O-Ag-doped ZnO 1wt%/perovskite and FTO/ZnO/H_2_O-ethanol mixtures-Ag-doped ZnO 1wt%/perovskite. According to Fig. 7, two characteristic diffraction peaks of the perovskite layer were observed at 14.91^◦^ and 28.38^◦^ that were attributed to (002) and (220).

Based on Fig. 7, the perovskite layer has high-intensity peaks in FTO/ZnO/H_2_O-ethanol mixtures-Ag-doped ZnO 1wt% bilayer ETL/perovskite. These high-intensity peaks indicate improved crystallinity of the perovskite layer for FTO/ZnO/H_2_O-ethanol mixtures-Ag-doped ZnO 1wt% bilayer ETL/perovskite. Furthermore, compared to the FTO/ZnO/H_2_O-ethanol mixtures-Ag-doped ZnO 1wt%/perovskite, the perovskite layer exhibited declining diffraction peaks in FTO/ZnO/H_2_O-Ag-doped ZnO 1wt%/perovskite, which confirmed the reduced crystal quality in Fig. 7.

### Optical properties

**Figure 8:**
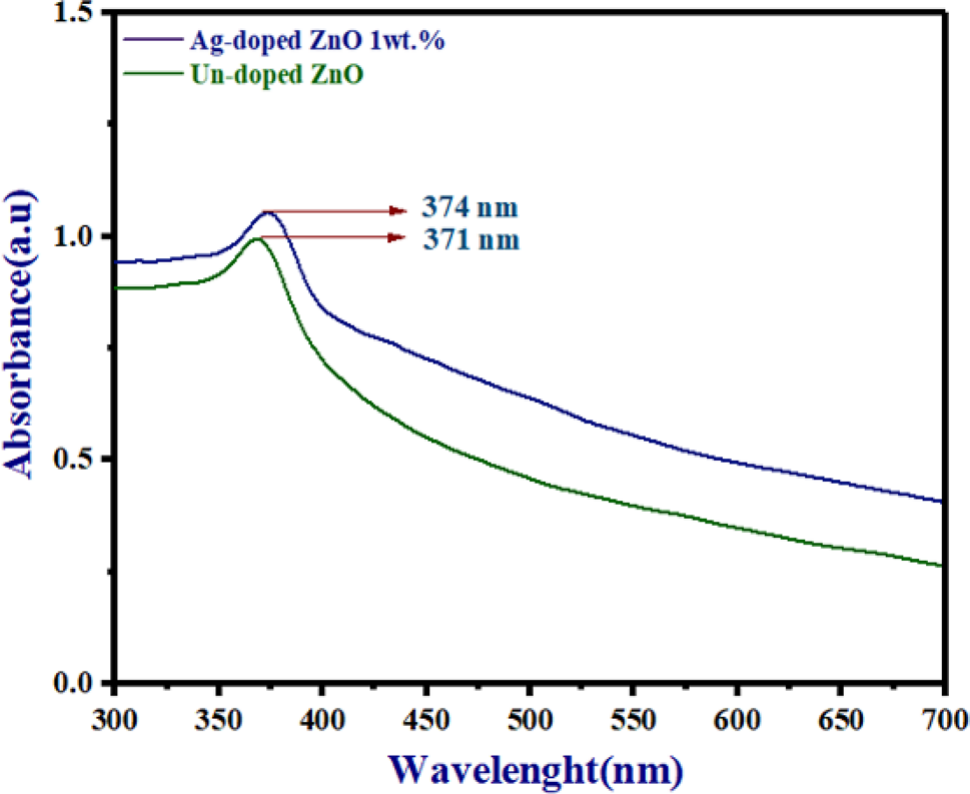
The absorbance spectra of un-doped ZnO and Ag-doped ZnO (at 1wt%) NPs.

Figure 8 shows absorption spectra for un-doped ZnO NPs and Ag-doped ZnO 1wt% NPs. The optical absorption peak of un-doped ZnO and Ag-doped ZnO 1wt% NPs between 371 and 374 nm. Ag-doped ZnO 1wt% improves absorbance as shown in Fig. 8. The absorption peak of Ag-doped ZnO 1wt% shifts to the visible light region (longer wavelengths) by creating defect states (shallow traps). According to the previous paper, with the Tauc method, the bandgaps of ZnO and Ag-doped ZnO at 1% are 3.06 eV and 3.03 eV respectively^36^.

**Figure 9:**
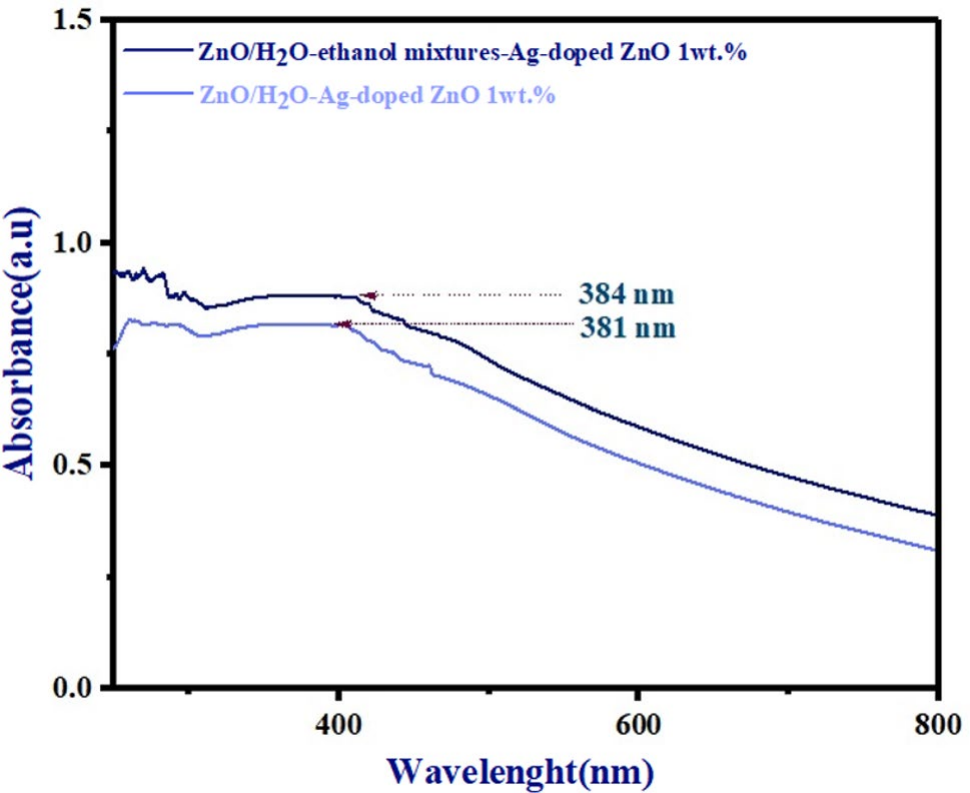
The absorbance spectra of both types of morphology for Ag-doped ZnO 1wt% buffer layer in ZnO/Ag-doped ZnO 1wt% bilayer ETL.

In addition, Fig. 9 indicates absorption spectra of glass/ZnO/H_2_O-Ag-doped ZnO 1wt% bilayer and glass/ZnO/H_2_O-ethanol mixtures-Ag-doped ZnO 1wt% bilayer ETLs respectively. According to Fig. 9, both types of morphology for Ag-doped ZnO 1wt% buffer layer in ZnO/Ag-doped ZnO 1wt% bilayer ETL have higher absorbance than un-doped ZnO and Ag-doped ZnO 1wt% ETLs. Based on the report of papers^35,36^, compared to ZnO/H_2_O-ethanol mixtures-Ag-doped ZnO 1wt% bilayer ETL in Fig. 9, the absorbance spectra of the un-doped ZnO and Ag-doped ZnO 1wt% ETLs was lower due to the increasing aggregation of NPs.

**Figure 10:**
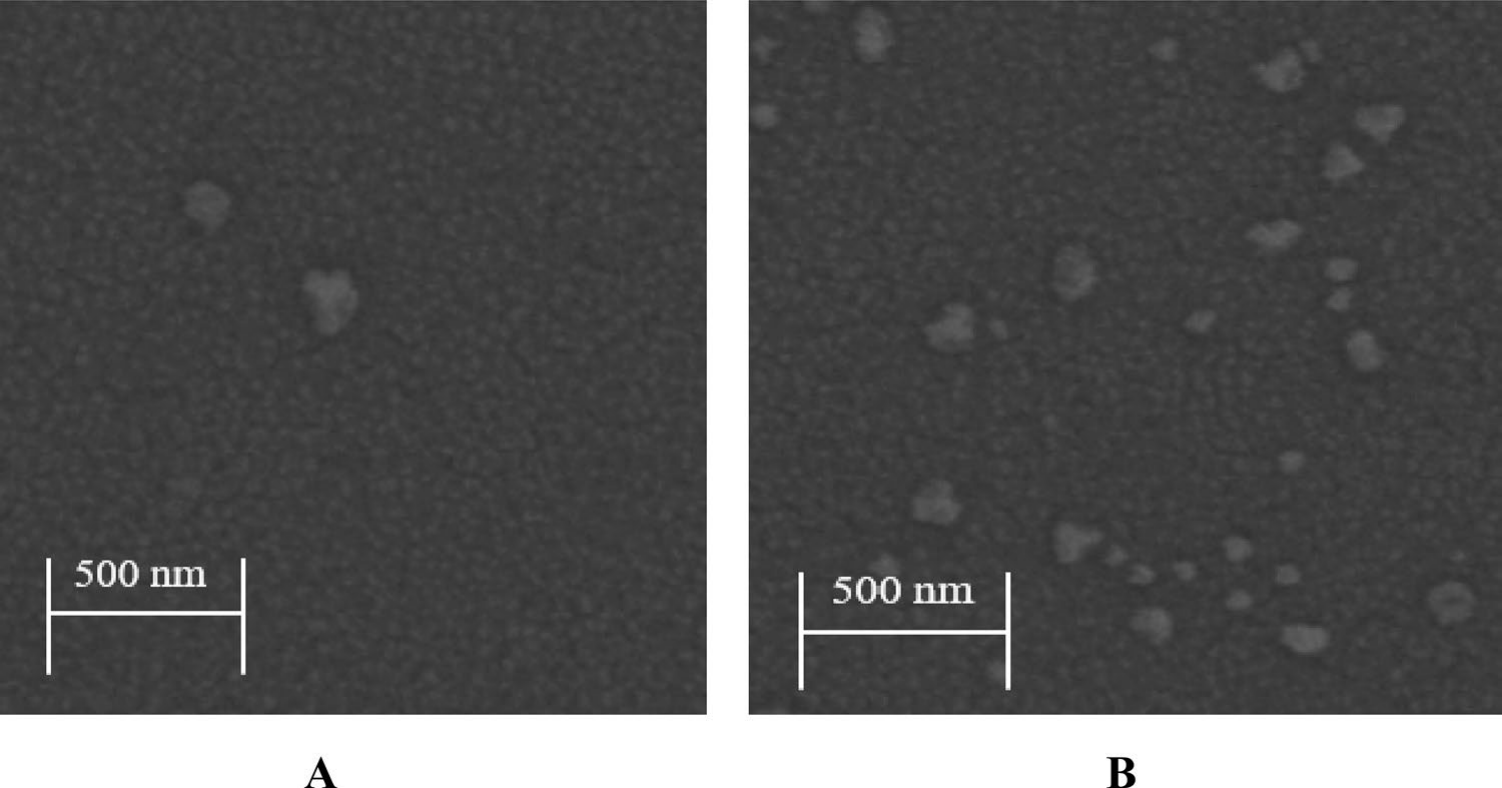
FE-SEM images of the ZnO/Ag-doped ZnO 1wt% bilayer ETL deposited by the Ag-doped ZnO 1wt% NPs dispersion in (**A**) H_2_O-ethanol mixtures, (**B**) H_2_O.

Additionally, in comparison to ZnO/H_2_O-ethanol mixtures-Ag-doped ZnO 1wt% bilayer ETL, the absorbance of ZnO/H_2_O-Ag-doped ZnO 1wt% bilayer ETL exhibits a slightly reduced, resulting from the uneven surface of ZnO/H_2_O-Ag-doped ZnO 1wt% bilayer ETL in Fig. 9. On the contrary, due to the uniform distribution of NPs, the ZnO/H_2_O-ethanol mixtures-Ag-doped ZnO 1wt% bilayer ETL has the highest absorbance (the absorption peak shifts toward the visible region) in Fig. 9. As a conclusion of the absorbance spectra findings, random aggregation of NPs could lead to an uneven surface, thereby decreasing the absorbance spectra of un-doped ZnO ETL, Ag-doped ZnO 1wt% ETL, and ZnO/H_2_O-Ag-doped ZnO 1wt% bilayer ETL. Moreover, the FE-SEM images show differences between the morphology of glass/ZnO/H_2_O-ethanol mixtures-Ag-doped ZnO 1wt% bilayer and glass/ZnO/H_2_O-Ag-doped ZnO 1wt% bilayer ETLs in Fig. 10A,B. The H_2_O-ethanol mixture-Ag-doped ZnO 1wt% NPs buffer layer prevents aggregating of Ag-doped ZnO 1wt% NPs (Fig. 10A). In contrast, Ag-doped ZnO 1wt% NPs dispersion in the H_2_O exhibited some random aggregating of NPs on ZnO/Ag-doped ZnO 1wt% bilayer ETL, leading to reduced optical properties (Fig. 10B).

FE-SEM results are consistent with the absorbance spectra in Fig. 9.

### Mott–Schottky (MS) analysis

The determination of the flat band potential of the ZnO/Ag-doped ZnO 1wt% bilayer ETL involved the Mott-Schottky analysis within a voltage bias range of − 0.5 to 0.1 V.

At the interface between the electrolyte and ETL, the capacity was calculated using Eq. (1)^41^:

1 $$\frac{1}{{C^{2} }} = \frac{2}{{\varepsilon \mathop \varepsilon \limits_{ \circ } qA^{2} N_{d} }}\left( {V - V_{f} - \frac{KT}{e}} \right)$$

**Figure 11:**
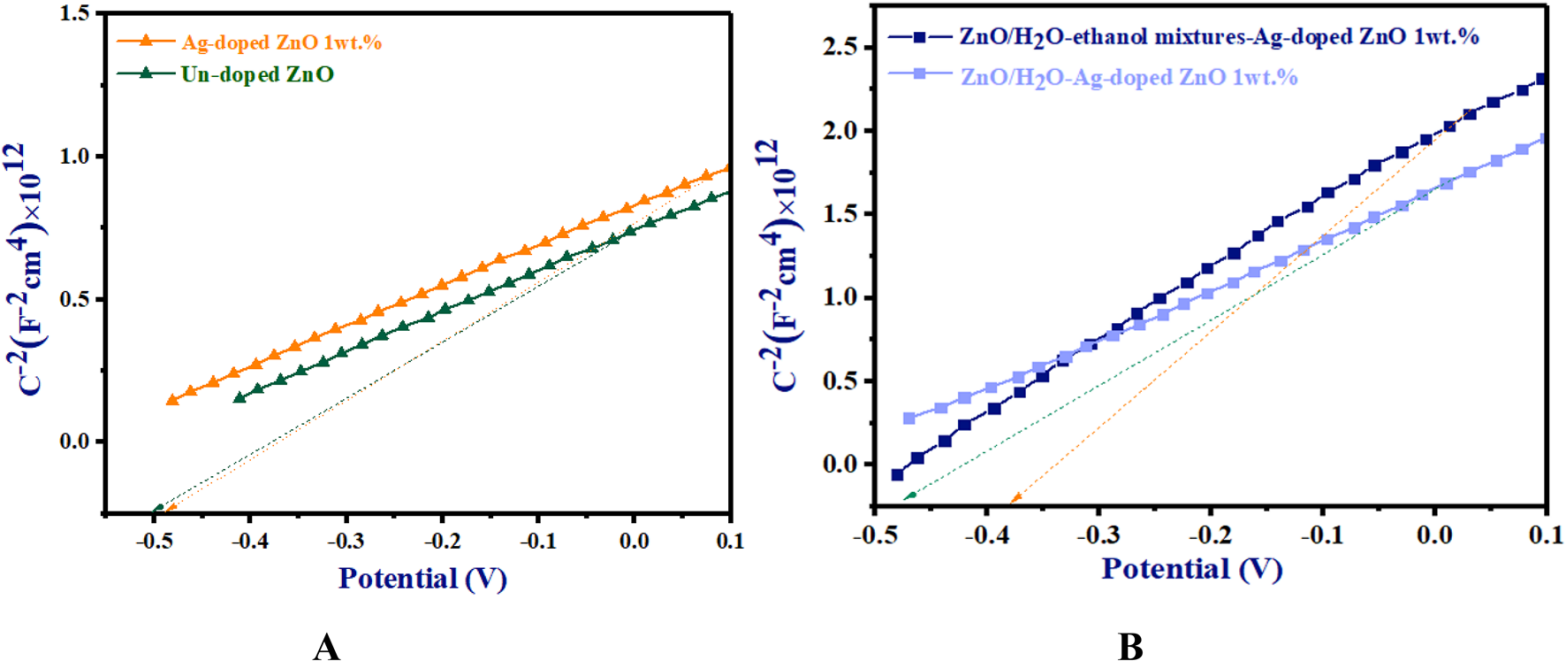
Mott-Schottky plots of (**A**) FTO/un-doped ZnO ETL and FTO/Ag-doped ZnO 1wt% ETL, and (**B**) FTO/ZnO/H_2_O-ethanol mixtures-Ag-doped ZnO 1wt% bilayer ETL and FTO/ZnO/H_2_O-Ag-doped ZnO 1wt% bilayer ETL.

In Eq. (1), (*C*) shows capacity, elementary charge (*q*) shows elementary charge, (*ɛ*) shows dielectric constant, (*N*_*d*_) shows doping density, (*V*) shows applied potential, (*V*_*f*_) shows flat band potential, (*A*) shows interface surface area and *T* and *K* show the temperature and the Boltzmann constant respectively. The Mott-Schottky plot is represented as $\frac{1}{{C}^{2}}$ vs. applied voltage (*V*), which determines the flat band potential. This is achieved through linear extrapolation of the linear portion, followed by subtracting the slope from the linear portion^42^. The positive slope represents n-type semiconductor materials. According to Fig. 11A, FTO/un-doped ZnO ETL and FTO/Ag-doped ZnO 1wt% ETL shows Mott-Schottky plots for n-type semiconductors. FTO/un-doped ZnO ETL and FTO/Ag-doped ZnO 1wt% ETL have built-in biases of -0.50 V and -0.49 V, respectively, in Fig. 11A. Accordingly, compared to un-doped ZnO ETL, shallow traps reduce the recombination rate slightly in Ag-doped ZnO 1wt% ETL. As shown in Fig. 11B, the Mott-Schottky plots of FTO/ZnO/H_2_O-ethanol mixtures-Ag-doped ZnO 1wt% and FTO/ZnO/H_2_O-Ag-doped ZnO 1wt% bilayer ETLs indicate n-type semiconductor. Based on the best fit in the linear portion of Mott-Schottky plots, ZnO/H_2_O-Ag-doped ZnO 1wt% and ZnO/H_2_O-ethanol mixtures-Ag-doped ZnO 1wt% bilayer ETLs have built-in biases (V_bi_) of -0.47 V and -0.36 V respectively in Fig. 11B. As the results, ZnO/H_2_O-ethanol mixtures-Ag-doped ZnO 1wt% and ZnO/H_2_O-Ag-doped ZnO 1wt% bilayer ETLs are better covered by dense pores, which prevent direct contact between the FTO and the perovskite layer. In comparison to other ZnO ETLs, ZnO/H_2_O-ethanol mixtures-Ag-doped ZnO 1wt% bilayer ETL shows the highest V_bi_, which indicates a lower charge recombination rate due to the reduced random aggregation of NPs and the smoother surface of Ag-doped ZnO 1wt% ETL.

### PL analysis

**Figure 12:**
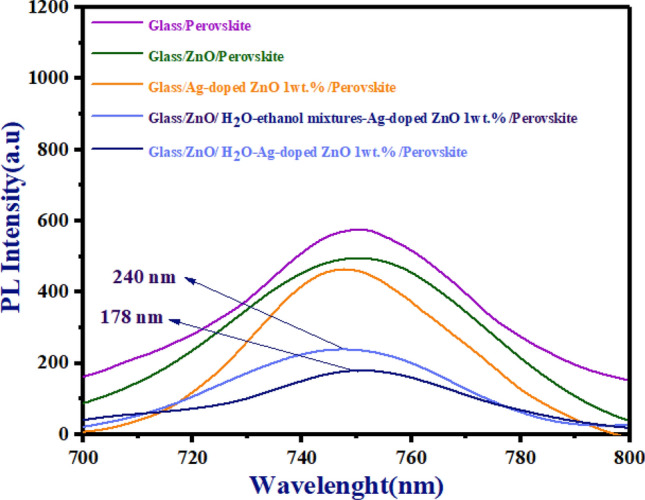
PL spectra of glass/perovskites, glass/un-doped ZnO/perovskite, and glass/Ag-doped ZnO 1 wt%/perovskite, glass/ZnO/H_2_O-ethanol mixtures-Ag-doped ZnO 1wt%/perovskite, and glass/ZnO/H_2_O-Ag-doped ZnO 1wt%/perovskite.

Figure 12 illustrates the PL spectra of glass/perovskites, glass/un-doped ZnO/perovskite, glass/Ag-doped ZnO 1wt%/perovskite, and glass/ZnO/H_2_O-ethanol mixtures-Ag-doped ZnO 1wt%/perovskite and glass/ZnO/H_2_O-Ag-doped ZnO 1wt%/perovskite.

We examined approaches to weak peak intensity of PL spectra for un-doped ZnO and Ag-doped ZnO at 1 wt% layers in the report of paper^36^. It should be noted that both types of morphology for Ag-doped ZnO 1wt% buffer layer in ZnO/Ag-doped ZnO 1wt% bilayer ETL have lower peak intensity than single-layer ETLs in Fig. 12. Here, the ZnO/Ag-doped ZnO 1wt% bilayer ETL demonstrates a lower recombination rate, compared to single-layer ETLs in Fig. 12. Nevertheless, in comparison to ZnO/H_2_O-Ag-doped ZnO 1wt% bilayer ETL, the ZnO/H_2_O-ethanol mixtures-Ag-doped ZnO 1wt% bilayer ETL revealed a weaker peak intensity at 178 nm.

**Figure 13:**
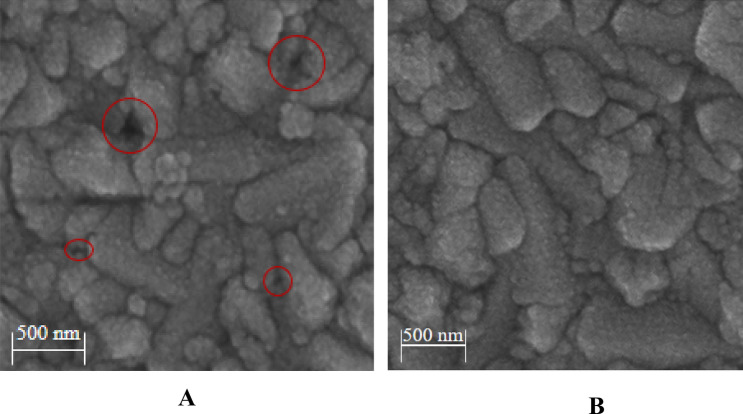
FE-SEM analyses (**A**) perovskite film deposited on the top of H_2_O-ethanol mixtures-Ag-doped ZnO 1wt% buffer layer, and (**B**) perovskite film deposited on top of the H_2_O-ethanol mixtures-Ag-doped ZnO buffer layer.

To understand these differences in PL spectra, Fig. 13A,B show FE-SEM of perovskites deposited on both types of morphology for Ag-doped ZnO 1wt% buffer layer. Moreover, Fig. 13A,B compare the wettability of the ZnO/Ag-doped ZnO 1wt% bilayer ETLs. As a result of the increased wettability in ZnO/H_2_O-Ag-doped ZnO 1wt% bilayer ETL, Fig. 13A indicates some defects in the perovskite layer. These defects lead to weak peak intensity in PL spectra. Furthermore, with low wettability in the ZnO/H_2_O-ethanol mixtures-Ag-doped ZnO 1wt% bilayer ETL, the H_2_O-ethanol mixtures-Ag-doped ZnO 1wt% contributes to the formation of a high-quality perovskite layer with low defects in Fig. 13B.

### J–V characteristics

**Figure 14:**
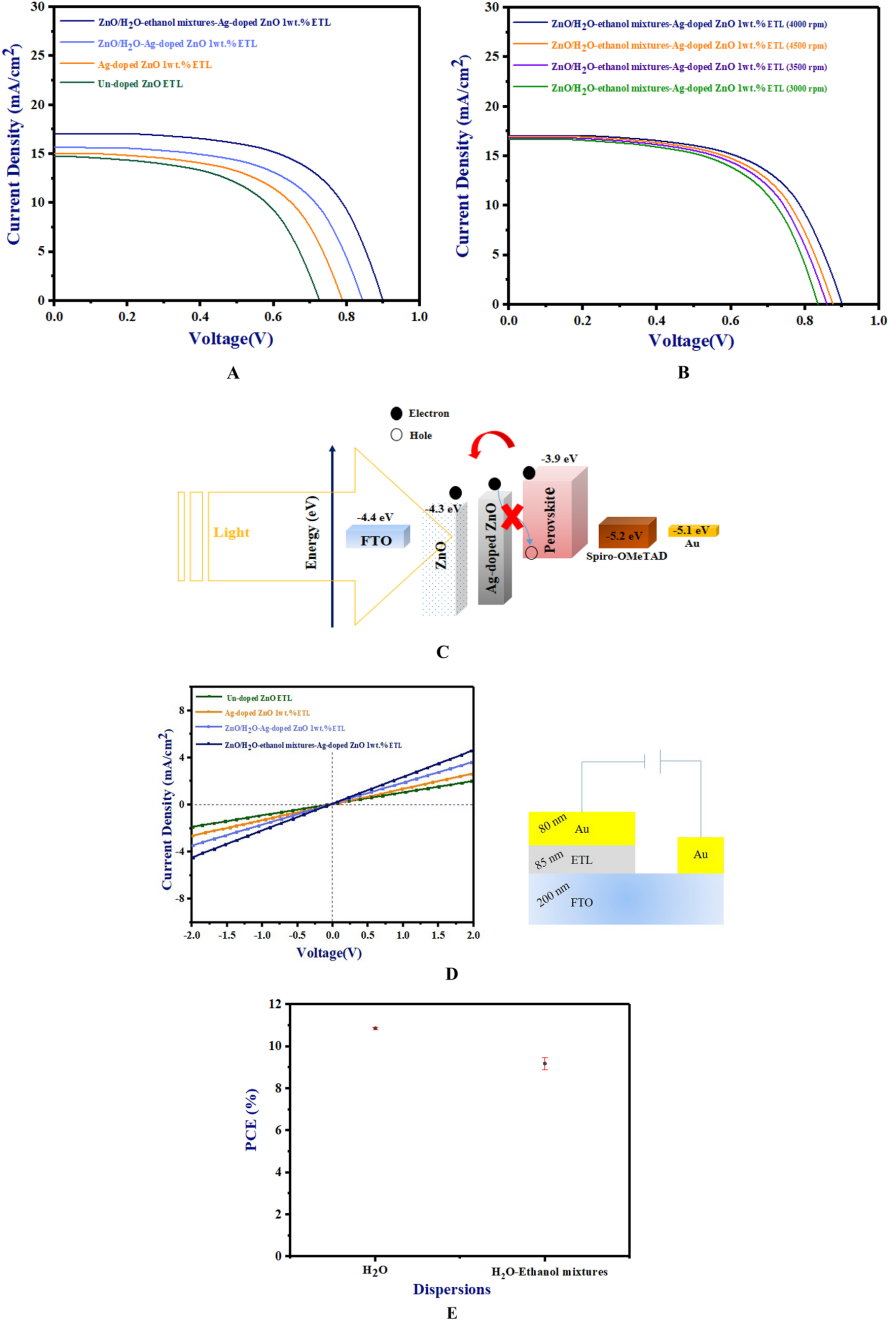
(**A**) J–V curves of un-doped ZnO ETL, Ag-doped ZnO ETL, and both types of morphology for Ag-doped ZnO 1wt% buffer layer in ZnO/Ag-doped ZnO 1wt% bilayer ETL-based planar PSCs, (**B**) J–V curves of ZnO/H_2_O-ethanol mixtures-Ag-doped ZnO 1wt% bilayer ETL-based PSC with different spinning speeds (at 3000 rpm, 3500 rpm, 4000 rpm, and 4500 rpm for 20 s) for deposition of H_2_O-ethanol mixtures-Ag-doped ZnO 1wt% buffer layer, (**C**) a schematic of the energy level diagram in ZnO/Ag-doped ZnO 1wt% bilayer ETL-based planar PSCs, (**D**) J–V curves of un-doped ETL, Ag-doped ZnO 1wt% ETL, ZnO/H_2_O-Ag-doped ZnO 1wt% bilayer ETL, and ZnO/H_2_O-ethanol mixtures-Ag-doped ZnO 1wt% bilayer ETL devices in the dark and schematic of the device, and (**E**) error bars of PCE (%) for ZnO/H_2_O-ethanol mixtures-Ag-doped ZnO 1wt% bilayer ETL-based planar PSC.

The J–V measurements were performed using an artificial solar simulator. The J-V curves of un-doped ZnO ETL-based PSC, Ag-doped ZnO 1wt% ETL-based PSC and both types of morphology for Ag-doped ZnO 1wt% buffer layer in ZnO/Ag-doped ZnO 1wt% bilayer ETL-based PSCs were observed in Fig. 14A. Moreover, Table 3 displays a summary of the J-V characteristics, including the open-circuit voltage (V_OC_), the fill factor (FF), the short-circuit current (J_SC_), and PCE (%) of un-doped ZnO ETL-based PSC, Ag-doped ZnO 1wt% ETL-based PSC, and both types of morphology for Ag-doped ZnO 1wt% buffer layer in ZnO/Ag-doped ZnO 1wt% bilayer ETL-based PSCs.

**Table 3:** J–V Characteristics of un-doped ZnO ETL, Ag-doped ZnO ETL, and both types of morphology for Ag-doped ZnO 1wt% buffer layer in ZnO/Ag-doped ZnO 1wt% bilayer ETL-based planar PSCs.

ETL	Buffer layer	J_sc_ (mA/cm^2^)	V_oc_	FF	η (%)
*Un-doped ZnO^36^	–	14.71 ± 0.05	0.73 ± 0.01	0.61	6.59 ± 0.05
*Ag-doped ZnO 1wt%^36^	–	15.01 ± 0.03	0.78 ± 0.01	0.62	7.40 ± 0.08
Un-doped ZnO	H_2_O–Ag-doped ZnO	15.44 ± 0.1	0.82 ± 0.005	0.66	9.18 ± 0.3
Un-doped ZnO	H_2_O-ethanol mixtures–Ag-doped ZnO	17.05 ± 0.05	0.90 ± 0.005	0.74	10.86 ± 0.05

*Other paper.

In the report of papers^35,36^, these reports demonstrated that Ag doping at 1wt% can lead to the creation of shallow traps in un-doped ZnO ETL. In Fig. 14A, due to the formation of these shallow traps, the Ag-doped ZnO 1wt% ETL achieves a higher conduction band maximum (C_BM_) than un-doped ZnO ETL, which results in greater charge carrier separation, a reduction in recombination rate, and increased charge carrier extraction^36^. As shown in Table 3, Ag doping at 1wt% improves V_oc_ (by reduction in recombination rates), J_sc_ (by increasing charge carrier extraction), FF (by achieving a higher (C_BM_) than un-doped ZnO ETL), and PCE (%) in Ag-doped ZnO 1wt% ETL-based PSCs. Based on Table 3, the PCE (%) of the ZnO/Ag-doped ZnO 1wt% bilayer ETL-based PSCs fabricated using different morphology of Ag-doped ZnO 1wt% buffer layer (Ag-doped ZnO NPs 1wt% dispersions in H_2_O-ethanol mixes and H_2_O) is higher than un-doped ZnO ETL-based PSC and Ag-doped ZnO 1wt% ETL-based PSC. In comparison with un-doped ZnO and Ag-doped ZnO 1 wt% ETLs, the surface of the ZnO/H_2_O-Ag-doped ZnO 1wt% bilayer ETL is better covered by dense pores, which leads to the prevention of direct contact between the FTO and perovskite layer. According to the results, the charge carrier extraction increases with reducing recombination rate in ZnO/H_2_O-Ag-doped ZnO 1wt% bilayer ETL-based PSC in Fig. 14A.

On the other hand, compared to ZnO/H_2_O-ethanol mixtures-Ag-doped ZnO 1wt% bilayer ETL, the reduction of PCE (%) for ZnO/H_2_O-Ag-doped ZnO 1wt% bilayer ETL-based PSC could be explained by a higher wettability of the H_2_O-Ag-doped ZnO 1wt% layer. With a high wettability of the H_2_O-Ag-doped ZnO 1wt% buffer layer, irregular grain sizes and random defects were observed in the perovskite layer for ZnO/H_2_O-Ag-doped ZnO bilayer ETL-based PSC.

The low crystal quality in the perovskite layer led to high recombination rate (reduced V_OC_ and FF), and reduced charge carrier extraction (reduced J_SC_) in ZnO/H_2_O-Ag-doped ZnO bilayer ETL-based PSC. As shown in Table 3, the ZnO/H_2_O-Ag-doped ZnO 1wt% bilayer ETL–based PSC achieved Jsc, V_oc_, FF, and PCE (%) values of 15.44 (mA/cm^2^), 0.82 (V), 0.66, and 9.18%, respectively.

In contrast, compared to un-doped ZnO ETL, Ag-doped ZnO 1wt% ETL, and ZnO/H_2_O-Ag-doped ZnO bilayer ETL in Table 2 and report of the paper^36^, random aggregation of NPs was reduced in ZnO/H_2_O-ethanol mixtures-Ag-doped ZnO 1wt% bilayer ETL. This reduction of random aggregation led to the formation of an even surface in ZnO/H_2_O-ethanol mixtures-Ag-doped ZnO 1wt% bilayer ETL. Hence, the absorption peak of ZnO/H_2_O-ethanol mixtures-Ag-doped ZnO 1wt% bilayer ETL shifts towards the visible region (longer wavelengths). According to the results, The J_SC_ improves with increasing light absorption in ZnO/H_2_O-ethanol mixtures-Ag-doped ZnO 1wt% bilayer ETL–based PSC in Table 3. In addition, due to the low wettability of ZnO/H_2_O-ethanol mixtures-Ag-doped ZnO 1wt% bilayer ETL, the perovskite layer has a high quality and few defects in ZnO/H_2_O-ethanol mixtures-Ag-doped ZnO 1wt% bilayer ETL–based PSC. Based on the results, further modification of the ZnO/H_2_O-ethanol mixtures-Ag-doped ZnO 1wt% bilayer ETL with low wettability leads to improved formation of the perovskite layer.

According to Fig. 14A, ZnO/H_2_O-ethanol mixtures-Ag-doped ZnO 1wt% bilayer ETL–based PSC exhibited an increase of 10.86% in PCE (%). This high PCE (%) can be attributed to several contributing factors. These factors include the enhancement of perovskite crystallinity due to a reduction in wettability of ZnO/H_2_O-ethanol mixtures-Ag-doped ZnO 1wt% bilayer ETL, an improvement in the interfacial contact of ZnO/perovskite (increased V_OC_ and FF), the enhancement of charge carrier extraction (by increasing light absorption) and reducing the recombination rate (by shallow traps and preventing direct contact between the FTO and the perovskite layer) in ZnO/H_2_O-ethanol mixtures-Ag-doped ZnO 1wt% bilayer ETL–based PSC.

Moreover, optimizing the thickness of the Ag-doped ZnO 1wt% buffer layer is the most important step in improving the performance of PSCs. Increasing the thickness of the Ag-doped ZnO 1wt% buffer layer in the ZnO/Ag-doped ZnO 1wt% bilayer ETL will decrease light absorption by the perovskite layer, which results in a decrease in photocurrent density.

Figure 14B shows the J–V curve for ZnO/H_2_O-ethanol mixtures-Ag-doped ZnO 1wt% bilayer ETL-based PSC which has been deposited H_2_O-ethanol mixtures-Ag-doped ZnO 1wt% buffer layer using different spinning speeds (at 3000 rpm, 3500 rpm, 4000 rpm, and 4500 rpm for 20 s). In addition, Table 4 shows the J–V characteristics of ZnO/H_2_O-ethanol mixtures-Ag-doped ZnO 1wt% bilayer ETL-based PSC with different spinning speeds (at 3000 rpm, 3500 rpm, 4000 rpm, and 4500 rpm) for deposition of H_2_O-ethanol mixtures-Ag-doped ZnO 1wt% buffer layer. The best-performing ZnO/H_2_O-ethanol mixtures-Ag-doped ZnO 1wt% bilayer ETL-based PSCs prepared by optimizing the thickness of H_2_O-ethanol mixtures-Ag-doped ZnO 1wt% buffer layer at 4000 rpm.

**Table 4:** J–V characteristics of ZnO/H_2_O-ethanol mixtures-Ag-doped ZnO 1wt% bilayer ETL-based PSC with different spinning speeds (at 3000 rpm, 3500 rpm, 4000 rpm, and 4500 rpm for 20 s).

ETL	Buffer layer	(rpm)	J_sc_ (mA/cm^2^)	V_oc_	FF	η (%)
*Un-doped ZnO^36^	–	–	14.71 ± 0.05	0.73 ± 0.01	0.61	6.59 ± 0.05
Un-doped ZnO	H_2_O-ethanol mixtures–Ag-doped ZnO	3000	16.68 ± 0.02	0.83 ± 0.005	0.65	9.64 ± 0.03
Un-doped ZnO	H_2_O-ethanol mixtures–Ag-doped ZnO	3500	16.82 ± 0.03	0.88 ± 0.005	0.70	10.44 ± 0.1
Un-doped ZnO	H_2_O-ethanol mixtures–Ag-doped ZnO	4000	17.05 ± 0.05	0.90 ± 0.005	0.74	10.86 ± 0.05
Un-doped ZnO	H_2_O-ethanol mixtures–Ag-doped ZnO	4500	16.95 ± 0.02	0.88 ± 0.005	0.72	10.54 ± 0.08

*Other paper.

According to Table 4, the ZnO/H_2_O-ethanol mixtures-Ag-doped ZnO 1wt% bilayer ETL-based PSCs (with 4000 rpm for deposition of H_2_O-ethanol mixtures-Ag-doped ZnO 1wt%) showed Jsc, V_oc_, FF, and PCE (%) values of 17.05 (mA/cm^2^), 0.90 (V), 0.74%, and 10.86%, respectively. Figure 14C shows the energy level diagram for ZnO/Ag-doped ZnO at 1wt% bilayer ETL planar PSCs. CV data were obtained from various studies^43,44^. As shown in Fig. 14B, the C_BM_ of the Ag-doped ZnO at 1wt% as buffer layer was higher than un-doped ZnO, indicating that modification of the Ag-doped ZnO 1wt% buffer layer can reduce the recombination rate by the tuning level of ETL/perovskite interface.

Furthermore, we examined the electrical properties of these ETLs by measuring the J–V curves of FTO/ETL/Au devices. Figure 14D indicates J–V curves of un-doped ETL, Ag-doped ZnO 1wt% ETL, ZnO/H_2_O-Ag-doped ZnO 1wt% bilayer ETL, and ZnO/H_2_O-ethanol mixtures-Ag-doped ZnO 1wt% bilayer ETL devices in the dark and schematic of the device.

Additionally, Fig. 14D shows that the slope of the ZnO/H_2_O-ethanol mixtures-Ag-doped ZnO 1wt% bilayer ETL-based device is higher than other ZnO ETL devices, which implies the ZnO/H_2_O-ethanol mixtures-Ag-doped ZnO 1wt% bilayer ETL-based device has increased electrical conductivity. Accordingly, we computed the electrical conductivity (σ) using the J–V curves^45^. The electrical conductivity (σ) values were 1.59 × 10^–3^ S per centimeter (S/cm), 2.61 × 10^–3^ S/cm, 2.96 × 10^–3^ S/cm, and 3.43 × 10^–3^ S/cm for un-doped ETL, Ag-doped ZnO 1wt% ETL, ZnO/H_2_O-Ag-doped ZnO 1wt% bilayer ETL, and ZnO/H_2_O-ethanol mixtures-Ag-doped ZnO 1wt% bilayer ETL devices, respectively. Increasing the electrical conductivity can be attributed to the improvement in electron mobility and interfacial contacts in the ZnO/H_2_O-ethanol mixtures-Ag-doped ZnO 1wt% bilayer. Improvement of interfacial contact of ZnO/H_2_O-ethanol mixtures-Ag-doped ZnO 1wt% bilayer could explain the low defect level in perovskites.

**Figure 15:**
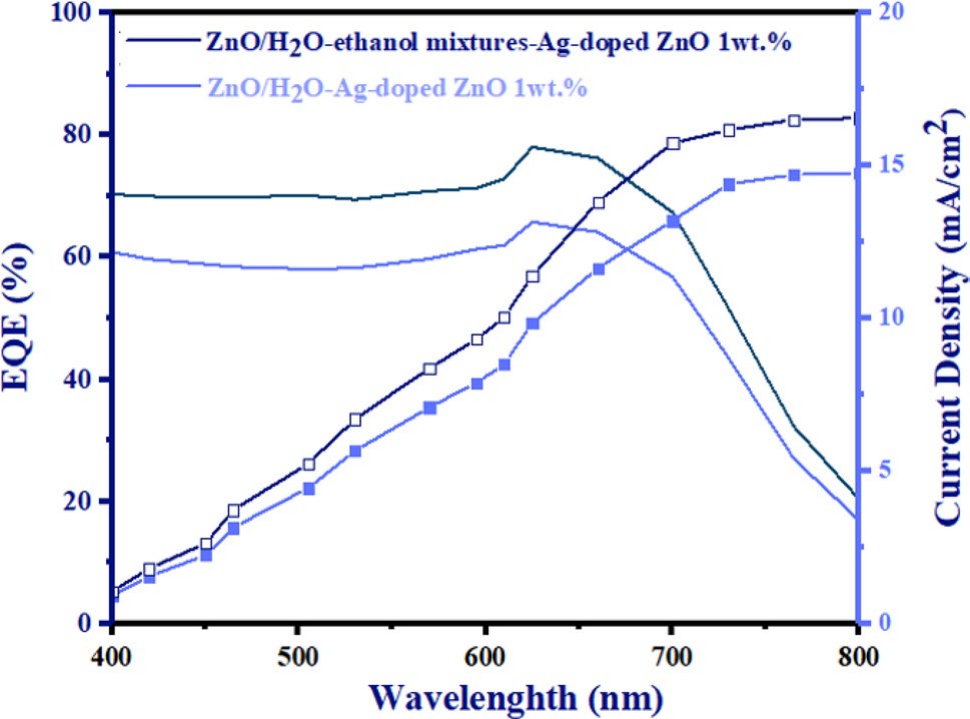
The EQE spectra of both types of morphology for Ag-doped ZnO 1wt% buffer layer in ZnO/Ag-doped ZnO 1wt% bilayer ETL-based PSCs.

**Figure 16:**
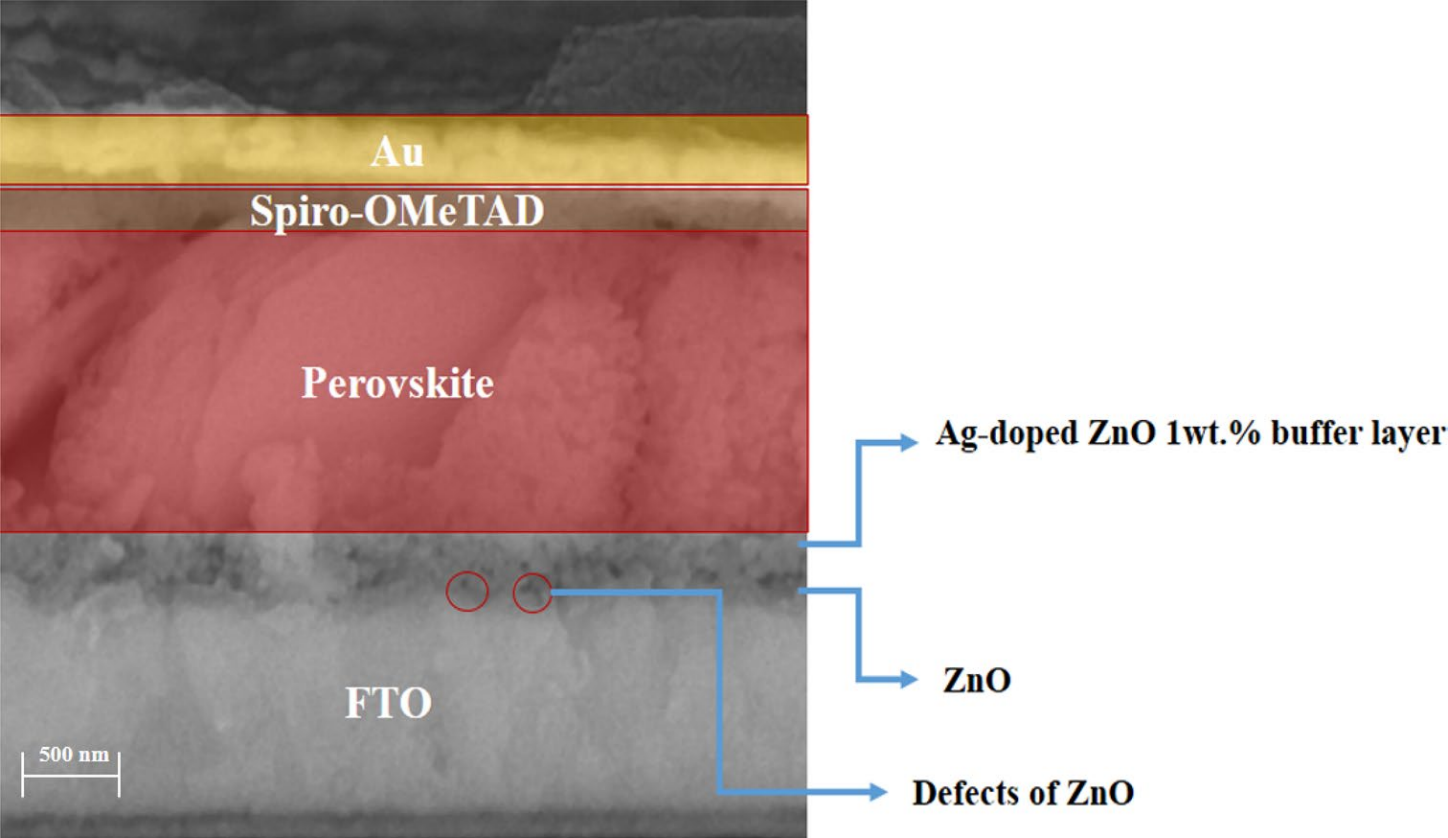
Cross-sectional FE-SEM of ZnO/H_2_O-ethanol mixtures-Ag-doped ZnO 1wt% bilayer ETL-based planar PSC.

Figure 14E depicts the PCE (%) of error bars with red, representing the standard deviation of 20 PSC devices. The EQE spectra of both types of morphology for Ag-doped ZnO 1wt% buffer layer in ZnO/Ag-doped ZnO 1wt% bilayer ETL-based PSCs are indicated in Fig. 15. According to Fig. 15, the J_sc_ values for ZnO/H_2_O-ethanol mixtures-Ag-doped ZnO 1wt% bilayer ETL–based PSC and ZnO/H_2_O-Ag-doped ZnO 1wt% bilayer ETL–based PSC were 16.80 mA/cm^2^ and 14.90 mA/cm^2^, respectively. Based on the EQE results, J-V measurements of bilayer ETL-based PSCs are reliable. Figure 16 shows cross-sectional FE-SEM of ZnO/H_2_O-ethanol mixtures-Ag-doped ZnO 1wt% bilayer ETL-based planar PSC. In Fig. 16, defects of the un-doped ZnO ETL are shown with red circles, which are covered by the H_2_O-ethanol mixtures-Ag-doped ZnO 1wt% buffer layer.

### Stability analysis

**Figure 17:**
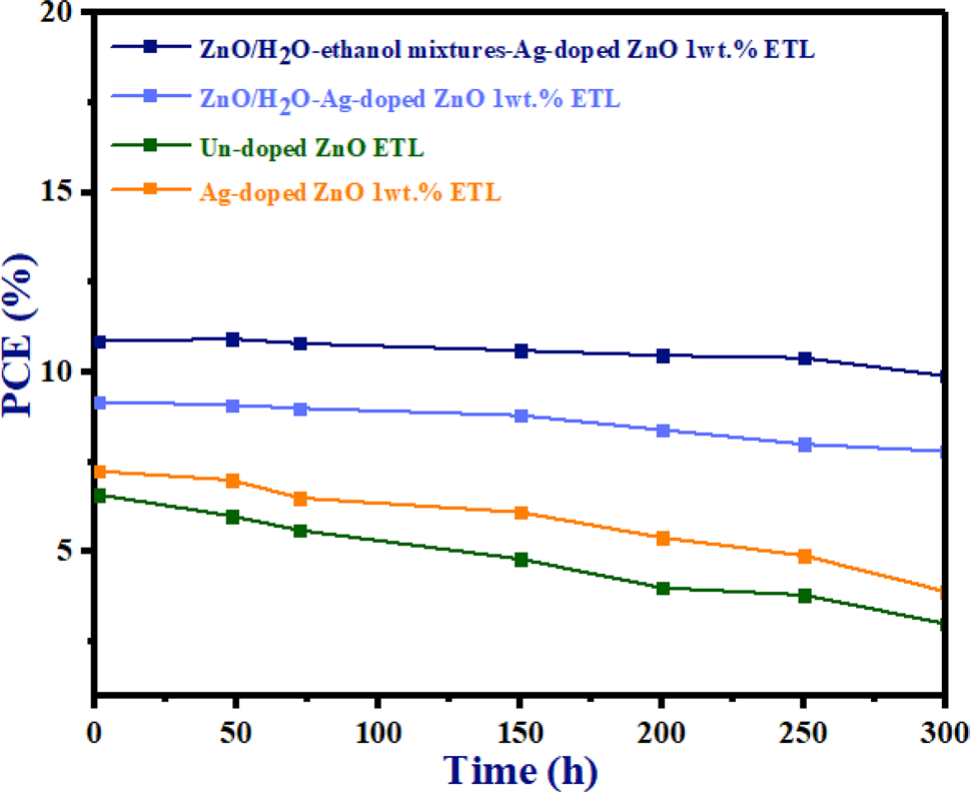
The PCE stability test versus time of un-doped ETL, Ag-doped ZnO 1wt% ETL, ZnO/H_2_O-Ag-doped ZnO 1wt% bilayer ETL, and ZnO/H_2_O-ethanol mixtures-Ag-doped ZnO 1wt% bilayer ETL based-PSCs.

For practical applications, the long-term stability of PSCs is essential, especially in ambient air. To examine the stability, based on Fig. 17, the PCE (%) of un-doped ETL, Ag-doped ZnO 1wt% ETL, ZnO/H_2_O-Ag-doped ZnO 1wt% bilayer ETL, and ZnO/H_2_O-ethanol mixtures-Ag-doped ZnO 1wt% bilayer ETL based-PSCs were continuously monitored in ambient air (humidity level of 35%) at 25°C for 300 h without encapsulation. The PCE (%) of un-doped ZnO ETL-based PSC declined after 16 h. However, the PCE (%) of Ag-doped ZnO 1wt% ETL-based PSC declined on the first day. Due to the defects passivation at the ZnO/perovskite interface by Ag doping, Ag- Ag-doped ZnO 1wt% ETL-based PSC has higher stability than un-doped ZnO ETL-based PSC. On the other hand, as shown in Fig. 17, compared to un-doped ZnO ETL and Ag-doped ZnO ETL-based PSCs, both types of morphology for Ag-doped ZnO 1wt% buffer layer in ZnO/Ag-doped ZnO 1wt% bilayer ETL**-**based planar PSCs display better stability**.** The improved stability of both types of morphology for Ag-doped ZnO 1wt% buffer layer in ZnO/Ag-doped ZnO 1wt% bilayer ETL-based planar PSCs is due to better coverage by bilayer ETLs in PSCs**.**

According to Fig. 17, ZnO/H_2_O-ethanol mixtures-Ag-doped ZnO 1wt% bilayer ETL enhances the environmental stability of un-doped ZnO ETL-based PSCs. In Fig. 17, ZnO/H_2_O-ethanol mixtures-Ag-doped ZnO 1wt% bilayer ETL-based-PSC maintains 94% of its original PCE (10.86%). Due to the smooth surface and low wettability, the ZnO/H_2_O-ethanol mixtures-Ag-doped ZnO 1wt% bilayer ETL improves perovskite crystallinity, thereby reducing degradation rates of PSCs. In contrast, compared to ZnO/H_2_O-ethanol mixtures-Ag-doped ZnO 1wt% bilayer ETL in Fig. 17, ZnO/H_2_O-Ag-doped ZnO 1wt% bilayer ETL led to the decline of PCE (%) by the uneven surface of Ag-doped ZnO 1wt% NPs. Additionally, the high wettability of ZnO/H_2_O-Ag-doped ZnO 1wt% bilayer ETL leads to the formation of defects on the perovskite layer, which accelerates the degradation of ZnO/Ag-doped ZnO 1wt% bilayer ETL-based PSCs.

To further investigate the stability of ZnO/H_2_O-ethanol mixtures-Ag-doped ZnO 1wt% bilayer ETL-based planar PSC, we performed a set of tests under constant temperature (at 50 °C for 100 h without encapsulation) and relative humidity (RH = 50% for 100 h without encapsulation).

**Figure 18:**
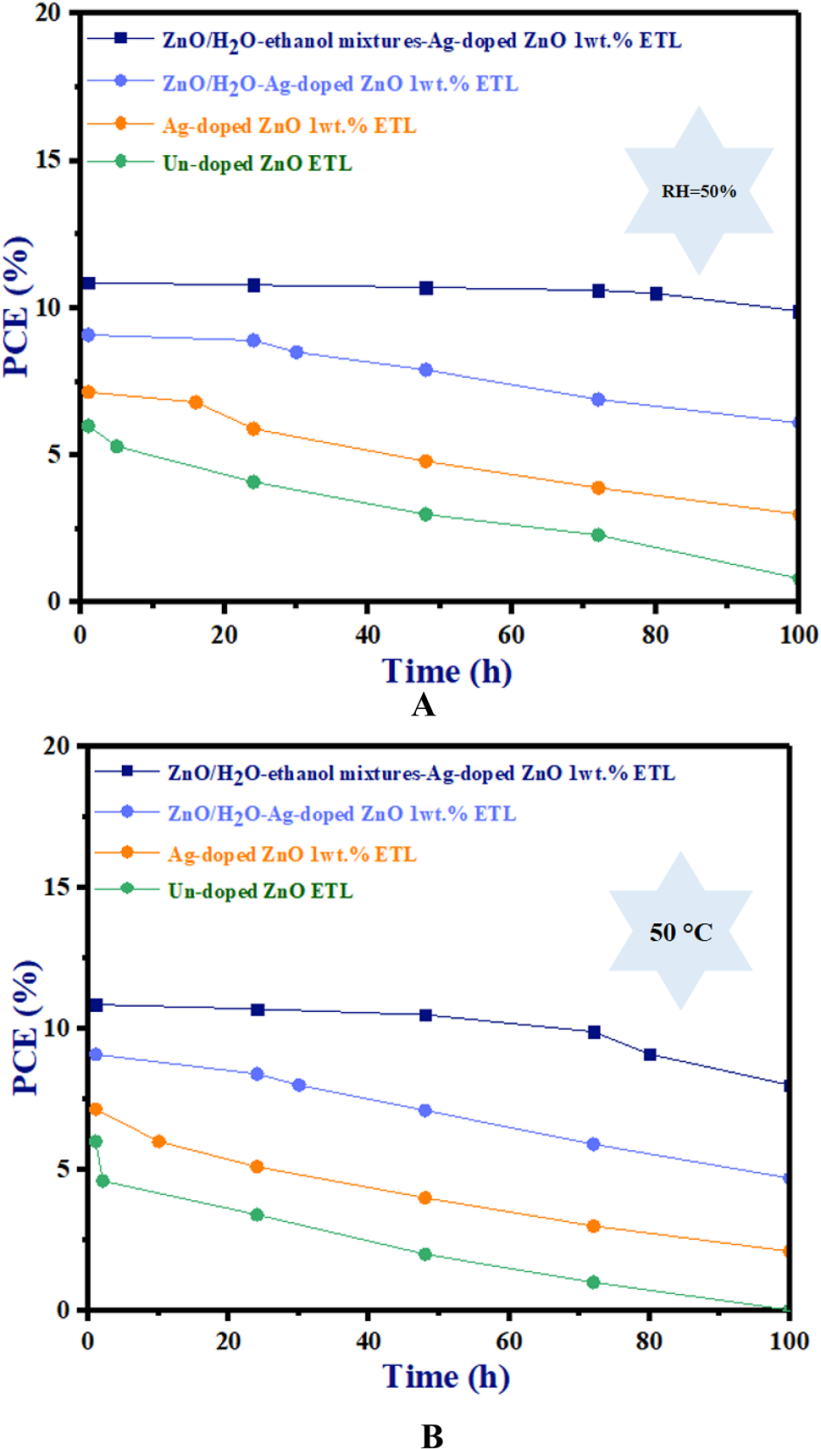
The PCE stability test versus time of un-doped ETL, Ag-doped ZnO 1wt% ETL, ZnO/H_2_O-Ag-doped ZnO 1wt% bilayer ETL, and ZnO/H_2_O-ethanol mixtures-Ag-doped ZnO 1wt% bilayer ETL based-PSCs under (**A**): 50% relative humidity, and (**B**): under thermal testing at 50 °C in the glove box.

It should be noted that the PCE (%) of PSCs under thermal testing was performed in a glovebox at 50°C. Figure 18A shows the operation of un-doped ETL, Ag-doped ZnO 1wt% ETL, ZnO/H_2_O-Ag-doped ZnO 1wt% bilayer ETL, and ZnO/H_2_O-ethanol mixtures-Ag-doped ZnO 1wt% bilayer ETL devices under humidity conditions (RH = 50%). Under humidity conditions (RH = 50%), the PCE (%) of un-doped ETL, Ag-doped ZnO 1wt% ETL, ZnO/H_2_O-Ag-doped ZnO 1wt% bilayer ETL-based PSCs dramatically decline after 5h, 17 h, and 30 h respectively.

In contrast, after 80 h, under humidity conditions (RH = 50%), the PCE (%) of ZnO/H_2_O-ethanol mixtures-Ag-doped ZnO 1wt% bilayer ETL-based PSC declines with a lower slope than other ETLs. As a result of the constant thermal stability test (at 50 °C) in Fig. 18B, compared to other ZnO ETL devices, ZnO/H_2_O-ethanol mixtures-Ag-doped ZnO 1wt% bilayer ETL-based PSC demonstrated decent thermal stability for 80 h. While, the PCE (%) of un-doped ETL, Ag-doped ZnO 1wt% ETL, ZnO/H_2_O-Ag-doped ZnO 1wt% bilayer ETL-based PSCs significantly decrease after 2 h, 10 h, and 24 h respectively in Fig. 18B.

As a result of constant temperature testing, the degradation mechanism is different from the degradation mechanism of humidity testing. These degradation mechanisms (such as phase transition of perovskite) are different between thermal and humidity testing due to the distinct environmental and chemical factors.

Consequently, the high quality of the perovskite layer formation, the better crystallinity, the compact contact between the un-doped ZnO and perovskite layer, and the smooth surface morphology of ZnO/H_2_O-ethanol mixtures-Ag-doped ZnO 1wt% bilayer ETL contribute to the improved thermal and moisture stability of un-doped ZnO ETL based-PSCs.

## Conclusions

In this paper, Ag-doped ZnO 1wt% was examined as a buffer layer to improve the PCE (%) and long-term stability in un-doped ZnO-based planar PSCs. Here, we proposed a simple method for controlling the morphology of the Ag-doped ZnO 1wt% buffer layer. The morphology of the buffer layer (Ag-doped ZnO 1 wt%) can be modified by adding ethanol to the dispersion of Ag-doped ZnO 1 wt% NPs in DI water for ZnO/Ag-doped ZnO 1wt% bilayer ETL. Furthermore, the low wettability of H_2_O-ethanol mixtures-Ag-doped ZnO as a buffer layer contributed to the formation of the perovskite layer with low defects in ZnO/Ag-doped ZnO 1wt% bilayer ETL-based PSC. The optimization of ZnO/H_2_O-ethanol mixtures-Ag-doped ZnO 1wt% bilayer ETL led to the fabrication of PSCs with PCE (%) of 10.86%, V_oc_, J_sc_, and FF values of 0.90 V, 17.05 mA/cm^2^, and 0.74, respectively. This improvement of PCE (%) can be attributed to the enhancement of perovskite crystallinity, improving charge carrier extraction of bilayer ETL, and better interfacial contact between un-doped ZnO ETL and perovskite layer in the ZnO/H_2_O-ethanol mixtures-Ag-doped ZnO 1wt% bilayer ETL-based PSCs. Moreover, the ZnO/H_2_O-ethanol mixtures-Ag-doped ZnO 1wt% bilayer ETL-based PSCs exhibit greater long-term stability than ZnO/H_2_O-Ag-doped ZnO 1wt% bilayer ETL-based PSCs in ambient air (by maintaining 10.70%). Consequently, this novel approach improves the performance and environmental stability of ZnO-based PSCs.

## Data Availability

Datasets will be available upon request, contact Ghazaleh Bagha at: gh.bagha@iau-tnb.ac.ir.
